# Genomic and phenotypic insights into the first imported monkeypox virus clade Ia isolate in China, 2025

**DOI:** 10.3389/fpubh.2025.1618022

**Published:** 2025-07-09

**Authors:** Chunhong Yin, Yan Li, Qing Duan, Dali Xu, Chengyunxiao Li, Ti Liu, Shujun Ding, Zhe Zhang, Aihua Zhang, Lianchen Fu, Zengqiang Kou

**Affiliations:** ^1^Infectious Disease Prevention and Control Section, Shandong Center for Disease Control and Prevention, Jinan, Shandong, China; ^2^Shandong Provincial Key Laboratory of Intelligent Monitoring, Early Warning, Prevention and Control for Infectious Diseases, Jinan, Shandong, China; ^3^School of Public Health, Shandong Second Medical University, Weifang, Shandong, China; ^4^School of Public Health, Shandong First Medical University, Jinan, Shandong, China; ^5^Taian Center for Disease Control and Prevention, Taian, Shandong, China

**Keywords:** monkeypox virus, clade Ia, genomic characteristics, phylogeny, virus isolation

## Abstract

The 2022 multi-country outbreak of monkeypox virus (MPXV) sparked global health concerns, as cases emerged increasingly outside traditionally endemic regions. This outbreak, spanning 2022–2023, was primarily driven by the clade IIb strain. Subsequently, a surge of MPXV cases caused by clades Ia and Ib in the Democratic Republic of the Congo and other African countries has garnered heightened attention. Notably, clade Ib has recently been imported into China. In this study, we report the first identification and genetic characterization of a novel imported MPXV strain in China, from a patient returning from an endemic area. Whole-genome sequencing and phylogenetic analysis confirmed its close relationship to MPXV clade Ia, which has not been previously reported in China. The strain was successfully isolated using Vero-E6 cell inoculation. Compared with the reference MPXV clade I strain, the imported Ia strain exhibited substantial genomic divergence, with 67 mutations identified. Among them, 28 non-synonymous mutations were found in genes associated with host interaction and viral pathogenesis. This study highlights the critical role of genomic surveillance and rapid molecular diagnostics in monitoring MPXV transmission and evolution. The successful isolation of a clade Ia strain provides a valuable resource for future research on its pathogenicity and for the development of targeted therapeutics.

## Introduction

Monkeypox virus (MPXV) is a zoonotic virus belonging to the genus Orthopoxvirus and is the causative agent of mpox in humans. It is endemic to regions of West and Central Africa, with the first human case reported in 1970 in the Democratic Republic of the Congo (DRC) (1). Since its discovery, cases have been predominantly reported in rural and rainforest regions of the Congo Basin in DRC, its neighboring countries, and in West Africa, with occasional exported cases reported outside Africa linked to travel or animal importation from endemic regions (2). However, the 2022 global outbreak marked a significant epidemiological shift, with widespread human-to-human transmission documented in over 100 non-endemic countries, raising concerns about sustained global transmission (3–6).

MPXV is genetically classified into two major clades: clade I (Congo Basin/Central African clade) and clade II (West African clade) (7, 8). Cameroon is the only known country where both clades co-circulate, with clade I and clade II predominating in the eastern and western regions, respectively (9). Clade II is further divided into subclades IIa and IIb, the latter being responsible for the 2022 global outbreak (10). Recent genomic surveillance in eastern DRC identified a genetically distinct lineage within clade I, now referred to as clade Ib (11). Earlier phylogenetic studies had already recognized multiple lineages within clade I, which are now collectively termed clade Ia (12, 13). Both clades are capable of causing mpox in humans; however, infections with clade II are typically milder and less transmissible compared to clade I infections (14). The 2022 clade IIb-driven outbreak, largely involving sexual transmission within networks of men who have sex with men, led to over 100,000 cases globally and prompted the World Health Organization (WHO) to declare mpox a Public Health Emergency of International Concern (PHEIC) (15–17). Although global case numbers have since declined, the outbreak remains ongoing (18). In contrast, the current outbreak in DRC has been linked to clade Ib, which is associated with more severe clinical presentations than clade II and has demonstrated potential for broader spread (11, 19). The detection of clade Ib cases in travelers returning to non-endemic regions further underscores the risk of international dissemination (20–22). In comparison, the epidemiological and molecular characteristics of clade Ia remain less well understood.

In this study, we report the identification and genomic characterization of an imported MPXV strain belonging to clade Ia, isolated from a returning traveler from the DRC to China. This marks the first documented importation of a clade Ia strain into China. Rapid detection and isolation of this case enabled timely public health intervention and appropriate clinical management. Whole-genome sequencing and phylogenetic analysis were conducted to explore the evolutionary relationships of this strain with other circulating MPXV variants. Thorough characterization of such imported strains is essential, as they may harbor unique genetic or phenotypic features with implications for public health. Furthermore, successful viral isolation provides a valuable resource for future research into MPXV pathogenesis, inter-clade differences, transmission mechanisms, and the development of antiviral therapies and vaccines.

## Materials and methods

### Ethics statement

This study was approved by Ethics Committee of Shandong Center for Disease Control and Prevention (Shandong CDC), and conducted in strict accordance with biosafety and ethical standards and the relevant laws and regulations of People’s Republic of China. Written informed consent was obtained from the patient for epidemiological investigations, clinical sample collection, diagnostic testing, virus isolation and data publication. All personal identifiers were removed to ensure confidentiality. The study posed no additional risks to the patient, and appropriate biosafety protocols were followed during sample handling and viral isolation. Experiments with live MPXV was performed under BSL-3 conditions at Shandong Center for Disease Control and Prevention, under the standard operating procedure approved by the Institutional Biosafety Committee.

### Sample collection and laboratory diagnosis

Blood samples and skin lesion swabs with vesicular fluid from different body sites were collected for subsequent laboratory diagnosis. Viral genome of the clinical samples was extracted using a commercial nucleic acid extraction kit (Bioperfectus, SDKF60101) following the manufacturer’s instructions. The MPXV genome was subjected to monkeypox virus genotyping detection, using a triplex real-time fluorescence quantitative PCR assay targeting clade Ia, clade Ib, and clade II MPXV provided by China CDC. The result interpretation criteria were as follows: No Ct value was considered negative, a Ct value ≤38 was considered positive, and a Ct value between 38 and 42 required retesting for further verification.

### Virus isolation and propagation

Vero E6 cells were used for viral isolation under BSL-3 containment. One day prior to inoculation, Vero E6 cells (1 × 10^6^ cells per well) were seeded into 6-well plate with 2 mL of culture medium. On the day of the assay, the cells were inoculated with clinical samples and incubated at 37°C in a humidified atmosphere with 5% CO₂ for 4 to 7 days. Cytopathic effects (CPE) were observed daily under light microscopy. Cell culture supernatants were collected at regular intervals for real-time PCR analysis. Following fixation with 4% paraformaldehyde (PFA), CPE was visualized and further confirmed by crystal violet staining.

### Whole-genome sequencing and phylogenetic analysis

Viral DNA was extracted using the QIAamp DNA Kit (Qiagen, 51,306) from clinical specimen. Subsequently, the MPXV whole genome was enriched from the extracted viral DNA using the Target Capture Kit for Mpox Virus (Baiyi Technology Co., Ltd., BK-MPXY024). Next-generation sequencing (NGS) was performed using the Illumina NextSeq2000 platform, generating paired-end reads. The complete genome was reference-based assembly using QIAGEN CLC Genomics Workbench (version23.0.3) (Supplementary material). Phylogenetic trees were constructed using IQ-TREE (version 2.4.0) with the maximum likelihood method. The newly imported strain was compared with MPXV genomes from the GISAID database.

## Results

### Clinical presentation of the reported monkeypox case

The first confirmed clade I monkeypox (MPXV-Ia) case in Shandong Province was a 34-year-old unmarried male, who worked overseas, in the Democratic Republic of the Congo (DRC) from January, 2024 to March, 2025. The patient initially developed perineal lesions, followed by fever, scattered skin rashes, and cervical lymphadenopathy. Initial treatment with antimalarial and antibiotic therapy did not lead to significant improvement. After returning to China, the patient sought medical attention due to persistent skin lesions. Based on clinical presentation and travel history, monkeypox infection was suspected and reported to the local CDC. The patient was subsequently hospitalized in an isolation ward, where the condition remained stable. He was born after the cessation of routine smallpox vaccination programs in China and had no history of smallpox or cowpox vaccination according to his self-report and medical history.

### Virus confirmation and isolation

**Figure 1:**
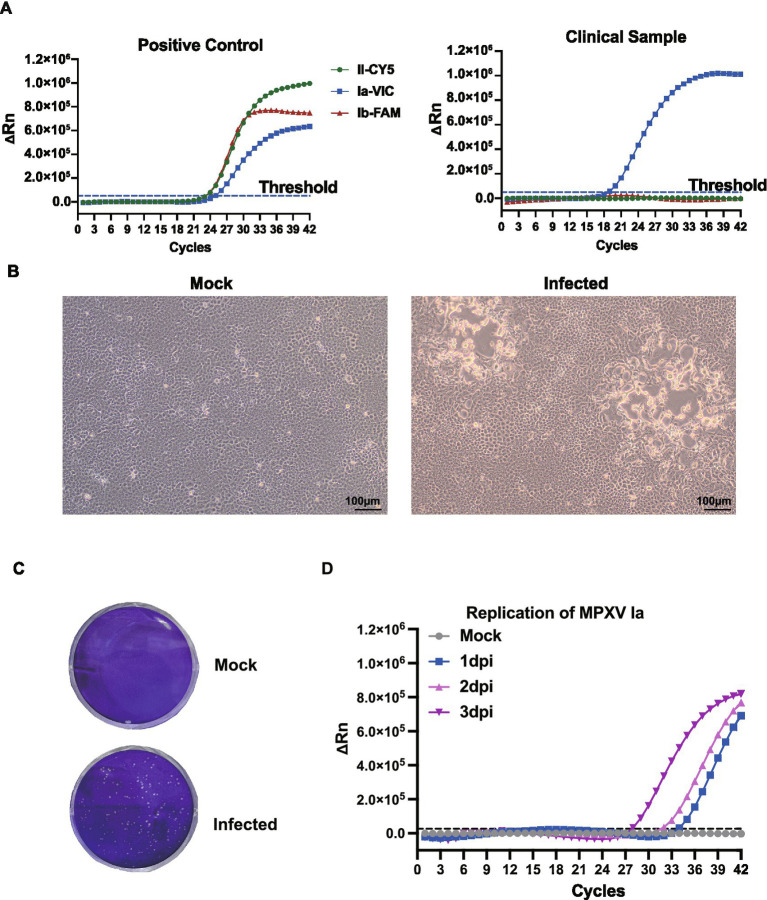
Identification and isolation of the imported MPXV clade Ia strain. **(A)** Development of a triplex real-time fluorescence quantitative PCR assay for the simultaneous detection of MPXV clades Ia, Ib, and II. The probe specific to clade II was labeled with Cyanine 5 (Cy5) at the 5′ end, while the probes specific to clades Ib and Ia were labeled with FAM and VIC, respectively. Clinical specimens from the patient were subjected to viral DNA extraction and subsequently analyzed using this assay. **(B)** Cytopathic effects (CPE) observed in Vero-E6 cells 3 days post-infection (dpi) with the isolated MPXV strain, visualized under light microscopy. **(C)** Plaque formation of the MPXV isolate on Vero-E6 cells, fixed and stained with crystal violet at 3 dpi. **(D)** Viral replication kinetics. Supernatants were collected at1, 2, and 3 dpi for DNA extraction and viral load quantification by real-time PCR. The average Ct values of the samples at 1, 2, 3 dpi were 34.2, 31.3, and 26.1.

The patient experienced fever, fatigue, and swollen lymph nodes, followed by vesicular skin lesions. Real-time PCR confirmed MPXV infection, with a cycle threshold (Ct) value of 18.8 in lesion swabs and 40.4 in serum, indicating a high viral load in skin lesions (Supplementary Table 1; Figure 1A). The lesion swab specimen, suspended in viral transport medium, was subsequently inoculated onto pre-seeded Vero-E6 cells. Cytopathic effects (CPE) were first observed on day 2 post-inoculation and became prominent by day 3, characterized by cell rounding, detachment, and cell death (Figures 1B,C). Daily collection of cell culture supernatants was performed for real-time PCR analysis. A progressive decrease in Ct values from day 1 (Ct = 34.2) to day 3 (Ct = 26.1) indicated successful viral replication and productive infection in Vero-E6 cells (Figure 1D).

### Genomic features and phylogenetic relationships

**Figure 2:**
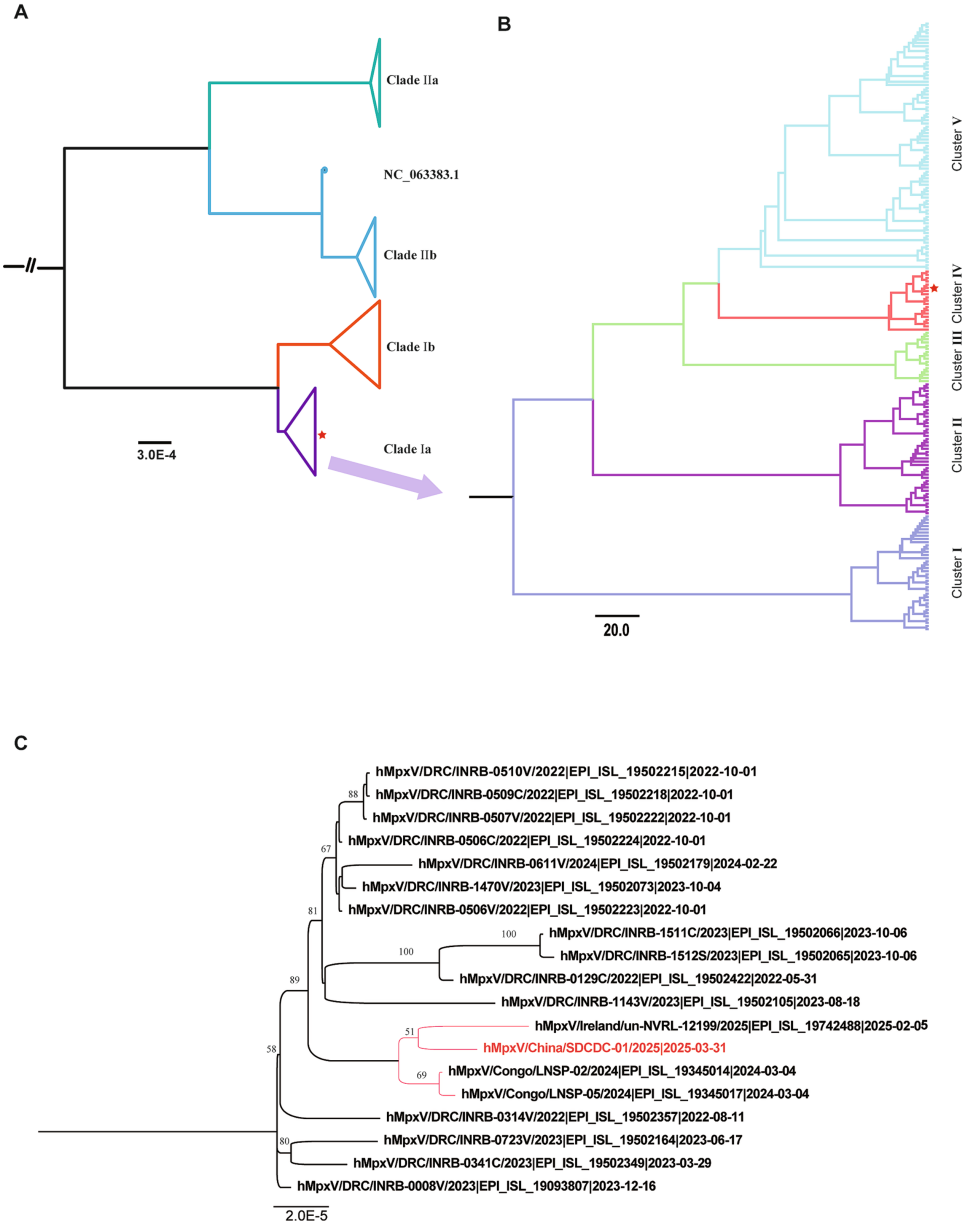
Phylogenetic analysis of MPXV genome sequences associated with the newly imported Mpox case in China. **(A)** A global phylogenetic tree incorporating the newly identified MPXV genome sequence alongside representative MPXV sequences available in the GISAID database. **(B)** Evolutionary classification of MPXV clade Ia into five distinct clusters. The newly identified isolate in this study belongs to Cluster IV. The red pentagram indicates the evolutionary branch of the imported strain in this study. **(C)** Detailed phylogenetic positioning of the MPXV isolate (hMpxV/China/SDCDC-01/2025|2025-03-31) within clade I, Cluster IV. The red-labeled text denotes the genome sequence identified in this study.

The complete genome of the imported MPXV strain post NGS analysis was 196,956 bp in length, with a GC content of 33% (details in Supplementary material). Phylogenetic analysis revealed that the virus sequence was closely related to MPVX clade Ia sequences (Figure 2A). Evolutionary analysis based on partial Ia sequences from the GISAID database revealed that all genomes were divided into five clusters (I-V), with the genome of this case belonging to Cluster IV (Figure 2B). Further analysis indicated that this genome shares high similarity with a sequence (EPI_ISL_19345014) submitted from the Republic of the Congo (Figure 2C). The genome of the Chinese strain shares a recent common ancestor with the Irish strain (EPI_ISL_19742488) within the Congo Basin clade Ia lineage, with a bootstrap support of 51, although it falls within the same broader cluster as strains from Congo Brazzaville.

**Figure 3:**
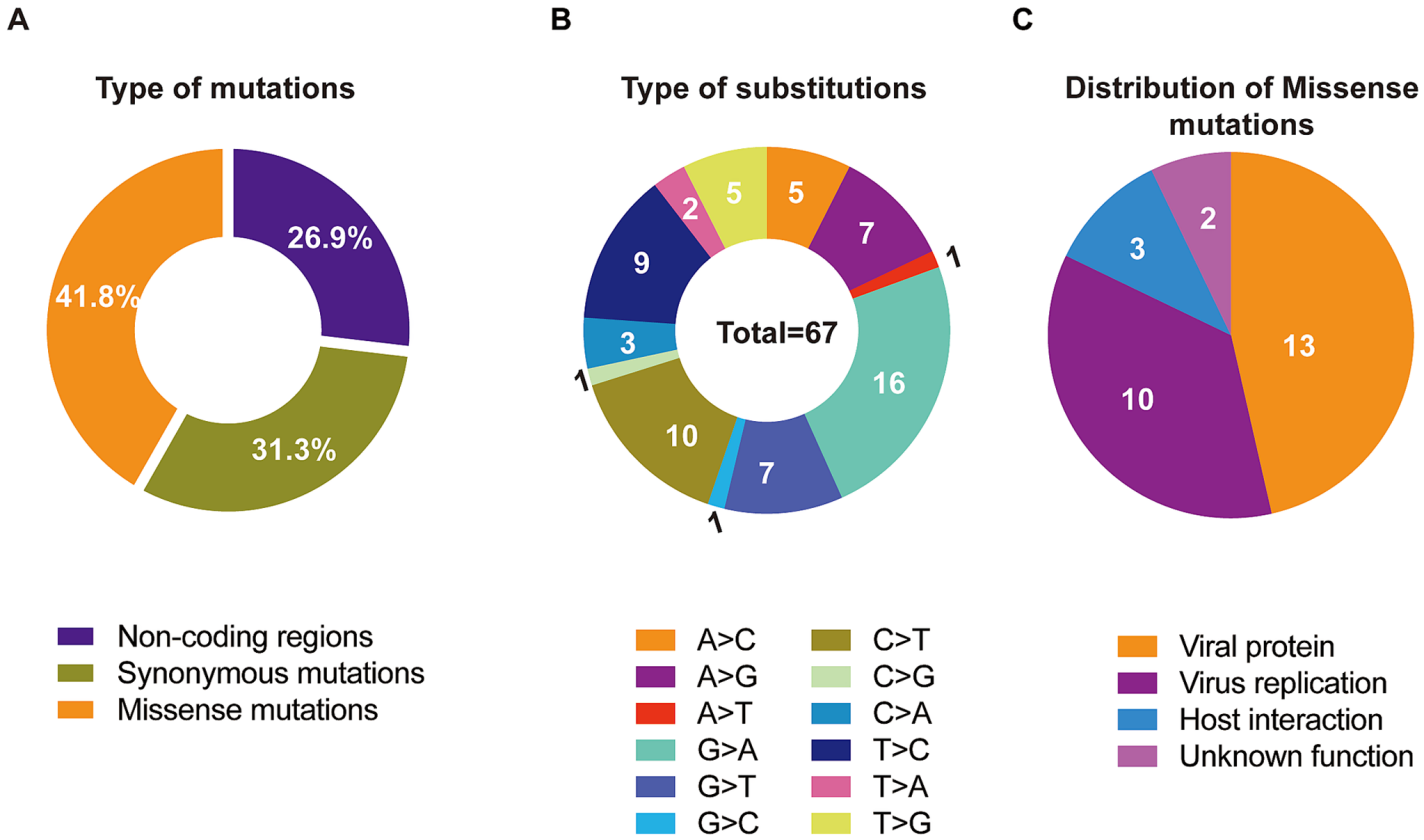
Molecular evolution characteristics of the imported MPXV clade Ia strain, in comparation to the clade I reference genome (GenBank Accession No. DQ011155.1). **(A)** Total ratio of 4 types of mutations across the identified MPXV clade Ia. **(B)** Summary of 12 types of substitutions across the whole genome of MPXV clade Ia. **(C)** Functional distribution of 28 missense mutations.

Single nucleotide polymorphisms (SNPs) are recognized as key drivers of rapid evolution and adaptive changes in poxviruses (23). Comparative genomic analysis with the clade I reference sequence (GenBank Accession No. DQ011155.1) identified 67 SNPs in the imported strain, including 28 missense mutations, 21 synonymous mutations, and the remainder located in non-coding regions of the genome (Table 1; Figure 3A). To further explore the underlying mechanisms of MPXV evolution, we analyzed the frequency of all 12 possible nucleotide substitution types across clade Ia genomes. The results revealed that C > T and G > A transitions were the most prevalent (Figure 3B), aligning with the known APOBEC3-mediated mutagenesis pathway previously implicated in MPXV evolution (6). Among the 28 non-synonymous mutations, 13 were located in genes encoding viral structural or functional proteins, 10 were associated with host interaction or immune modulation, 3 were involved in viral replication, and 2 had no clearly defined function (Figure 3C).

**Table 1:** Comparison of mutations between isolated Ia stain and clade I reference sequence DQ011155.1.

No.	Position	Type of substitutions	Gene	Mutation type	Aminao acid subsitution	Function
1	116	A > C		Non-coding regions		
2	186	A > C		Non-coding regions		
3	256	A > C		Non-coding regions		
4	956	G > A	OPG001	Synonymous mutations		Host interaction
5	3,355	G > T	OPG003	Missense mutations	L433I	Host interaction
6	6,111	A > G	OPG015	Missense mutations	V4A	Host interaction
7	6,611	T > G		Non-coding regions		
8	8,972	A > C		Non-coding regions		
9	11,487	C > T	OPG023	Missense mutations	C528Y	Host interaction
10	11,744	G > A	OPG023	Synonymous mutations		
11	12,801	G > A	OPG023	Missense mutations	A9V	
12	20,750	A > G		Non-coding regions		
13	21,755	T > C	OPG034	Synonymous mutations		Host interaction
14	26,495	C > T		Non-coding regions		
15	33,363	G > A	OPG048	Synonymous mutations		
16	34,597	C > T	OPG049	Missense mutations	E261K	Host interaction
17	36,113	G > C	OPG052	Missense mutations	S56T	Host interaction
18	36,485	C > T	OPG053	Missense mutations	D118N	Viral protein
19	37,213	G > A	OPG054	Synonymous mutations		
20	38,108	C > T	OPG054	Missense mutations	R12Q	Host interaction
21	39,376	G > T	OPG056	Missense mutations	T602N	Viral protein
22	44,265	C > A	OPG062	Synonymous mutations		
23	54,858	G > T	OPG071	Synonymous mutations		
24	58,394	G > A	OPG074	Synonymous mutations		
25	60,290	T > C	OPG077	Synonymous mutations		
26	60,853	G > A	OPG077	Missense mutations	P39S	Host interaction
27	63,368	G > A	OPG080	Synonymous mutations		
28	76,574	A > G	OPG094	Missense mutations	Q136R	Viral protein
29	83,912	T > C	OPG105	Synonymous mutations		
30	89,895	C > T	OPG109	Synonymous mutations		
31	92,017	G > A		Non-coding regions		
32	97,180	T > C	OPG115	Missense mutations	Y42H	Viral protein
33	103,045	C > G	OPG119	Missense mutations	T88R	Virus replication
34	103,508	T > C	OPG120	Missense mutations	H213R	Viral protein
35	110,647	A > G	OPG127	Missense mutations	Y193H	Virus replication
36	126,208	C > T	OPG144	Missense mutations	M41I	Viral protein
37	129,367	T > C	OPG148	Synonymous mutations		
38	129,368	G > T	OPG148	Missense mutations	A329S	Virus replication
39	134,829	G > A		Non-coding regions		
40	137,964	G > T		Non-coding regions		
41	138,454	T > A	OPG153	Missense mutations	D387V	Viral protein
42	139,000	C > T	OPG153	Missense mutations	R205H	Viral protein
43	140,136	A > C	OPG155	Missense mutations	I101R	Viral protein
44	143,272	T > C	OPG161	Synonymous mutations		
45	145,574	T > A	OPG165	Missense mutations	S39T	Unknown
46	146,212	A > T	OPG165	Synonymous mutations		
47	146,499	G > A		Non-coding regions		
48	149,682	T > G	OPG172	Missense mutations	C94W	Viral protein
49	150,101	A > G	OPG173	Missense mutations	I29V	unknown
50	151,725	G > A	OPG175	Synonymous mutations		
51	156,654	C > T	OPG181	Synonymous mutations		
52	156,883	A > G		Non-coding regions		
53	171,226	A > G	OPG199	Missense mutations	I142M	Host interaction
54	171,710	G > A	OPG199	Missense mutations	V304M	
55	178,124	T > C		Non-coding regions		
56	179,019	G > A		Non-coding regions		
57	180,349	G > T	OPG209	Synonymous mutations		
58	180,829	G > A	OPG209	Synonymous mutations		
59	183,985	C > A	OPG210	Synonymous mutations		
60	189,383	G > A	OPG005	Missense mutations	M131I	Host interaction
61	189,974	G > T		Non-coding regions		
62	190,857	T > C	OPG015	Missense mutations	V4A	Host interaction
63	193,613	C > A	OPG003	Missense mutations	L433I	Host interaction
64	196,012	C > T	OPG001	Synonymous mutations		
65	196,712	T > G		Non-coding regions		
66	196,782	T > G		Non-coding regions		
67	196,852	T > G		Non-coding regions		

## Discussion

This study presents the first identification, isolation, and genomic characterization of a monkeypox virus (MPXV) clade Ia strain imported into China. The detection of this case in a traveler returning from the Democratic Republic of the Congo (DRC) underscores the continuing risk of cross-border transmission of MPXV from endemic regions, particularly in light of the recent emergence and global spread of multiple clades. While the global mpox outbreak from 2022 to 2023 was largely attributed to clade IIb, the identification of clade Ia in this study provides important insights into the diversity of circulating MPXV lineages and their potential to cause disease beyond Africa.

The imported case described here illustrates the capacity of clade I strains to present with classical mpox symptoms, including fever, lymphadenopathy, and vesicular lesions. Although clade IIb has dominated the recent epidemiological landscape due to its efficient human-to-human transmission, clade I lineages, including both Ia and the recently defined Ib have long been associated with more severe clinical outcomes and higher mortality rates. Thus, the importation of a clade Ia strain into China is of significant concern, warranting heightened surveillance and preparedness, especially in regions with travel ties to endemic areas.

Regarding the potential transmission of the virus in the DRC, although the perineal distribution of lesions suggests the possibility of sexual transmission, the patient did not self-identify as a man who has sex with men (MSM) and denied any contact with commercial sex workers. However, due to the limitations of self-reported data and the retrospective nature of the investigation, the possibility of underreported sexual exposure cannot be ruled out. Additionally, the patient noted that several local colleagues in the DRC had taken sick leave due to febrile illnesses during the same period, raising the possibility of non-sexual transmission routes such as close physical contact or fomite-mediated exposure.

Phylogenetic analysis confirmed that the isolated strain belongs to clade Ia and is closely related to MPXV strains circulating in Congo-Brazzaville. However, the patient confirmed travel only within Kinshasa and surrounding regions in the DRC and explicitly denied visiting Congo-Brazzaville. Given the frequent cross-border population flow across the Congo River, it remains possible that the imported strain may reflect regional transmission dynamics involving viruses circulating in both countries. Comparative genomic analysis revealed 67 single nucleotide polymorphisms (SNPs) compared to the clade I reference genome (GenBank Accession No. DQ011155.1), including 23 non-synonymous mutations. These mutations occurred in genes involved in host interaction and viral production, such as ankyrin repeat (ANK) proteins, viral structural proteins and replication associated proteins, suggesting potential adaptive evolution that could influence viral fitness and pathogenicity. Notably, just five of these mutations were APOBEC3-like substitutions (TC > TT and GA > AA), consistent with the editing pattern previously observed for the Cla I mpox viruses from 2018 to 2022 (17, 24). The relatively low numbers of APOBEC3 mutations in clade Ia suggest its multiple zoonotic introductions.

Since the 2022 global outbreak, the clade IIb lineage B.1 has remained the dominant MPXV lineage in China (25–28). Until the end of 2024, all identified cases belonged to this clade, reflecting patterns of global transmission. However, the first case of MPXV clade Ib was reported as an imported case from the Democratic Republic of the Congo (DRC) in late 2024, marking the first documented introduction of this lineage into China (21). Globally, clade Ia remains relatively under-characterized, with limited genomic and phenotypic data available. This study contributes valuable new insights into clade Ia’s evolutionary dynamics, genetic diversity, and potential public health implications, enhancing our understanding of its role in the broader context of MPXV evolution and transmission.

Importantly, the successful isolation of the virus in Vero-E6 cells and observation of cytopathic effects (CPE) post-inoculation highlights its efficient replication capacity *in vitro*. This provides an indispensable resource for downstream research, including pathogenesis studies, antiviral drug screening, and vaccine development. Given the genetic divergence and observed mutations, functional studies will be critical to assess whether the clade Ia strain exhibits altered host range, transmission potential, or virulence compared to other clades. This case also highlights the critical importance of rapid molecular diagnostics and genomic surveillance in outbreak response and containment. The triplex qPCR assay targeting different MPXV clades enabled swift identification of the viral lineage, facilitating appropriate public health interventions and clinical management. As MPXV continues to evolve and spread, particularly through global travel, integration of real-time molecular typing into surveillance systems will be essential for early detection of emerging lineages.

In conclusion, the importation of a clade Ia MPXV strain into China expands the known geographical reach of this lineage and emphasizes the dynamic nature of MPXV transmission. Continued genomic surveillance, coupled with international collaboration, will be key to understanding the evolutionary trajectory and public health risks associated with all MPXV clades. Future studies should focus on characterizing the functional consequences of observed mutations and assessing the potential for clade I strains to establish sustained transmission chains in non-endemic settings.

## Data Availability

The whole-genome sequence of the Mpox virus clade Ia strain reported in this article has been deposited in the GISAID database under the accession number EPI_ISL_19867420, with the virus designated as hMpxV/China/SD-SDCDC-01/2025. The sequence is publicly accessible. For further inquiries, please contact the corresponding author.
